# Patient-reported outcome measures in pediatric solid organ transplantation: Exploring stakeholder perspectives on clinical implementation through qualitative description

**DOI:** 10.1007/s11136-020-02743-8

**Published:** 2021-01-14

**Authors:** Samantha J. Anthony, Katarina Young, Sarah J. Pol, Enid K. Selkirk, Tom Blydt-Hansen, Suzanne Boucher, Aviva Goldberg, Lorraine Hamiwka, Lotte Haverman, Joanna Mitchell, Simon Urschel, Maria Santana, Jennifer Stinson, Katie Sutherland, Lori J. West

**Affiliations:** 1 Hospital for Sick Children, 686 Bay Street, Toronto, ON M5G 0A4 Canada; 2 Factor-Inwentash Faculty of Social Work, University of Toronto, Toronto, Canada; 3 Canadian Donation and Transplantation Research Program, Edmonton, Canada; 4 School of Rehabilitation Science, McMaster University, Hamilton, Canada; 5 British Columbia Children’s Hospital, Vancouver, Canada; 6 Faculty of Medicine, University of British Columbia, Vancouver, Canada; 7 Health Science Centre Winnipeg, Winnipeg, Canada; 8 Max Rady College of Medicine, University of Manitoba, Winnipeg, Canada; 9 Alberta Children’s Hospital, Calgary, Canada; 10 Cumming School of Medicine, University of Calgary, Calgary, Canada; 11 Amsterdam UMC, University of Amsterdam, Emma Children’s Hospital, Psychosocial, Amsterdam, The Netherlands; 12 Stollery Children’s Hospital, Edmonton, Canada; 13 Department of Pediatrics, University of Alberta, Edmonton, Canada; 14 Lawrence S. Bloomberg Faculty of Nursing, University of Toronto, Toronto, Canada

**Keywords:** Patient-reported outcome measures, Qualitative, Pediatric, Solid organ transplantation, Patient engagement, Implementation

## Abstract

**Purpose:**

Patient-reported outcome measures (PROMs) are standardized instruments used to collect data about the subjective assessment of medical care from the patient perspective. Implementing PROMs within pediatric clinical settings has gained increasing importance as health services prioritize patient-centred pediatric care. This study explores the perspectives of pediatric solid organ transplant patients, caregivers, and healthcare practitioners (HCPs) on implementing PROMs into clinical practice.

**Methods:**

Qualitative description methods were used to elicit stakeholder perspectives. Semi-structured interviews were conducted across five Canadian transplant centres. Purposive sampling was used to obtain maximum variation across age, gender, and transplant program for all participants, as well as discipline for HCPs.

**Results:**

The study included a total of 63 participants [patients (*n* = 20), caregivers (*n* = 22) and HCPs (*n* = 21)]. Nearly all participants endorsed the implementation of PROMs to enhance pediatric transplant clinical care. Three primary roles for PROMs emerged: (1) to bring a transplant patient’s overall well-being into the clinical care conversation; (2) to improve patient communication and engagement; and, (3) to inform the practice of clinical pediatric transplant care. Insights for effective implementation included completing electronic PROMs remotely and prior to clinical appointments by patients who are eight to 10 years of age or older.

**Conclusions:**

This study contributes to current research that supports the use of PROMs in clinical pediatric care and guides their effective implementation into practice. Future directions include the development, usability testing, and evaluation of a proposed electronic PROM platform that will inform future research initiatives.

## Introduction

Patient-reported outcome measures (PROMs) are standardized instruments used to collect data about the subjective assessment of medical care from the patient perspective [1–4]. In this context, PROMs represent “any report of the patient’s health condition that comes directly from the patient, without interpretation of the patient’s response by a clinician or anyone else” [5] and can comprise information about symptoms, functional status, satisfaction, adherence and quality of life (QOL) [6–10]. When implemented effectively, PROMs can engage patients meaningfully and capture their varied experiences and attitudes [1–4]. Further, findings show that PROM data can increase patients’ satisfaction with care and potentially improve patients’ symptom management, overall well-being, and QOL [7, 11–13]. It is documented that the systematic collection of PROM data is more reflective of underlying health status than clinical objective reporting [7, 8, 11–13].

Efforts to implement PROMs into clinical practice are in the early stages of development [9], and research that considers the clinical application of PROMs in pediatric care is scarce [1, 14, 15]. However, implementing PROMs within pediatric settings has gained increasing importance as health services prioritize patient-centred care [1–4, 14, 16]. Research in adult clinical settings offers preliminary support for the role of PROMs in assessing the burden of disease and treatment and enhancing health outcomes through improved patient-provider communication, shared decision-making, and self-care management [7, 11–13, 17]. Emerging research indicates that the systematic integration of PROMs in pediatric care may result in increased patient-provider communication about psychosocial issues, such as social and emotional functioning [15, 18, 19], increased referrals to psychosocial supports [18], and improved health-related QOL [19]. These are meaningful outcomes that support the use of PROMs in pediatric patient populations that require a preventative approach to address risks associated with health-related QOL [19].

Nonadherence to life-long medical follow-up is a well-documented concern in pediatric transplant care, leading to potential consequences such as graft loss, organ rejection and death [20–22]. Pediatric transplant patients also have an increased risk of experiencing psychological distress, social isolation, impaired social functioning, cognitive difficulties, and behavioural problems [23–29]. Using PROMs within this patient population could identify concerns regarding functional and emotional status, help healthcare practitioners (HCPs) to detect under- and unrecognized problems (e.g., depression and anxiety), and solicit support from a multi-disciplinary team when necessary [18].

Integrating PROMs into clinical pediatric transplant care settings requires a shared vision among patients, caregivers and HCPs [2, 4, 30, 31]. The need to ‘build collaborative action’ among stakeholders is considered necessary to create a capacity for change in the process of implementation [32, 33]. Yet to date, research has not explored stakeholder perspectives on integrating PROMs into pediatric transplant care. The purpose of the current qualitative study is to elicit perceptions from patients, caregivers, and HCPs about the potential role of PROMs in the clinical care of pediatric transplant patients to inform effective implementation in this setting.

## Materials and methods

Qualitative description is a research method that assumes a naturalistic approach [34]. It strives to depict a phenomenon by staying close to the ‘surface’ meaning of words and provides a ‘comprehensive summary’ of events that reflects their occurrence with minimal interpretation [35]. Qualitative description was used in the current study to elicit stakeholder perspectives of those directly involved in implementing PROMs into clinical pediatric transplant care.

Participants were recruited across five of the largest pediatric transplant centres in Canada, each receiving institutional research ethics approval: Alberta Children’s Hospital (REB18-0140), British Columbia Children’s Hospital (H18-00,391), Health Sciences Centre Winnipeg (HS21697 (H2018:146)), Stollery Children’s Hospital (Pro00079805), and The Hospital for Sick Children (REB1000057043). Eligible patients included any solid organ transplant recipient between eight and 17 years of age, who was ≥ three months post-transplant. Eligible caregivers included any individuals who acted as the primary caregiver(s) for an eligible patient, even if the patient chose not to participate in the study. All patients and caregivers were required to be English-speaking with the capacity to participate in an interview as determined by a HCP in their circle of care. Eligible HCPs included any member of the interdisciplinary healthcare team who has worked within pediatric solid organ transplantation for a minimum of one year. Purposive sampling was used to obtain maximum variation across age, gender, and transplant program for all participants, as well as discipline for HCPs. Written informed consent or assent was obtained from all participants; when patients provided assent, informed consent was obtained from their parents/legal guardians. Demographics were collected for all study participants and selected medical data was collected for patient participants.

A semi-structured interview guide was developed by the research team based on clinical and research experience [36]. Interviews were approximately 45 minutes in length and were conducted in-person by two facilitators (KY & SJA) trained in qualitative methods. Questions explored participant perspectives about QOL and their experiences around the use of PROMs in clinical care. Two PROMs were reviewed with participants – the PedsQL™ Generic Core Scales [37] and the PedsQL™ Transplant Module [38]; their selection was based on a systematic review identifying PROMs used in pediatric solid organ transplantation [36]. After reviewing the PROMs, participants were shown a short video of a Dutch electronic PROM (ePROM) platform [39, 40], which illustrated the implementation of ePROMs into clinical use in a pediatric hospital.

Interviews were audio-recorded, transcribed verbatim, de-identified, and subjected to content and thematic analysis as outlined by Elo and Kynga [41]. Two research team members (SJP & KY) coded the data independently, and categories were reviewed and refined until consensus and thematic saturation were reached [41, 42]. Trustworthiness was achieved through soliciting rich description and facilitating member checking within interviews, as well as hosting frequent team meetings to support in-depth, iterative analysis with reflexive discussion among team members. The qualitative software program, N-Vivo 12 [43], was used for qualitative data management.

## Results

A total of 61 interviews were conducted with 63 participants–20 patients (PTs), 22 caregivers (CGs), and 21 HCPs. Three eligible patients/caregivers declined to participate due to scheduling conflicts, one declined due to disinterest, and all consenting participants completed the study. Two interviews comprised the patient and their parent due to patient preference. Participants represented a diverse sample across several variables, including ethnicity, geographic location, organ transplant program and socio-demographic background. Demographic characteristics for the sample are summarized in Table 1.

**Table 1:** Demographic characteristics of participants

Patients	
Female, *n* (%)	11/20 (55%)
Age at time of interview (yrs), median (range)	13.5 (9.0–17.0)
Transplant type, *n* (%)	
Heart	6 (30%)
Lung	1 (5%)
Liver	5 (25%)
Kidney	7 (35%)
Multi-organ	1 (5%)
Race/ethnicity, *n* (%)	
White	9 (45%)
Indigenous	4 (20%)
South Asian	2 (10%)
Black	1 (5%)
Filipino	1 (5%)
Somali	1 (5%)
South African	1 (5%)
Prefer not to say	1 (5%)

Caregivers	
Female, *n* (%)	18/22 (82%)
Age at time of interview (yrs), median (range)	47.0 (31.0–64.0)
Transplant type (patient), *n* (%)	
Heart	7 (31.8%)
Lung	1 (4.5%)
Liver	6 (27.1%)
Kidney	7 (31.8%)
Multi-organ	1 (4.5%)
Relationship to patient, *n* (%)	
Parent	20 (91%)
Grandparent	1 (4.5%)
Legal guardian	1 (4.5%)
Marital status, *n* (%)	
Married/common law	18(81.2%)
Separated/divorced	2 (9.1%)
Single	2 (9.1%)
Annual household income range, *n* (%)	
< 20,000	2 (9.1%)
20,000–39,000	1 (4.5%)
40,000–49,000	3 (13.6%)
60,000–69,000	4 (18.2%)
80,000–89,000	3 (13.6%)
> 100,000	7 (31.8%)
Prefer not to say	2 (9.1%)
Race/ethnicity, *n* (%)	
White	13 (59%)
Indigenous	3 (13.6%)
Filipino	2 (9.1%)
South Asian	2 (9.1%)
African	1 (4.5%)
Prefer not to say	1 (4.5%)

Healthcare practitioners	
Female, *n* (%)	16/21 (76%)
Years in transplantation at current institution, median (range)	13.0 (2.0–22.0)
Healthcare practitioner type, *n* (%)	
Physician	7 (34%)
Nurse practitioner	3 (14%)
Nurse	2 (9%)
Psychologist	2 (9%)
Social worker	2 (9%)
Dietician	1 (5%)
Occupational therapist	1 (5%)
Patient care coordinator	1 (5%)
Pharmacist	1 (5%)
Physical therapist	1 (5%)
Race/ethnicity, *n* (%)	
White	15 (72%)
South Asian	2 (9%)
Asian	1 (5%)
Black	1 (5%)
Prefer not to say	2 (9%)

Nearly all patients, caregivers and HCPs (60/63; 95%) endorsed the implementation of PROMs to enhance pediatric transplant clinical care. Three participants acknowledged the potential of PROMs to solicit essential patient information but were uncertain regarding their clinical use. Collectively, participants described three primary roles for PROMs that offered support for their use in pediatric clinical care: (1) to bring a transplant patient’s overall well-being into the clinical care conversation; (2) to improve patient communication and engagement; and, (3) to inform the practice of clinical pediatric transplant care. Insights gleaned for the effective implementation of PROMs are presented.

### The role of PROMs to bring pediatric transplant patients’ overall well-being into the clinical care conversation

Almost all participants supported the systematic integration of PROMs into clinical care in pediatric transplantation as it facilitated sharing a comprehensive view of a patient’s health with HCPs. Most participants highlighted a gap in clinical care whereby a consistent and formal assessment of a patient’s well-being and QOL is notably absent in clinical encounters. One caregiver noted: “At clinic [HCPs] are mainly focused on the ‘medical’ unless you tell them that there are other issues” (CG-1). A patient participant identified the role of PROMs to address this concern: “I feel like [PROMs] could uncover information that is otherwise not apparent to the medical team” (PT-3). One physician admitted: “We are often guilty of really only focusing on the medical problem and the treatment around the medical problem” (HCP-3). Another caregiver acknowledged the potential benefit of PROMs to affirm the “wholeness” (CG-2) of the patient experience by stating: “[Feeling] supported in the wholeness of the experience of the transplant would be beneficial–very validating” (CG-2). HCPs considered PROMs to be helpful in this regard, highlighting a specific potential for addressing mental health concerns: “It gives you a holistic view… ‘cause we don’t always focus on mental health” (HCP-4).

Three patients did not support the integration of PROMs into clinical care, yet still acknowledged that the instruments addressed topics of well-being that were not usually prioritized in physician–patient encounters. One patient indicated that they would rather talk about these concerns rather than complete a PROM: “[If asked to complete PROMs] I’d feel annoyed… I feel it’s easier to talk about the things” (PT-4). Two patients were older adolescents who explained that they did not support the use of PROMs in clinical care because they did not want to share information about their mental health.

Caregivers and HCPs articulated that the current system is “reactionary” (HCP-5) instead of “proactive”, relative to addressing mental health concerns through prevention. Using PROMs as a new, systematic approach to screening was supported. One HCP stated: “The only way to bring prevention into [clinical care] is to open up the discussion” (HCP-8). HCPs also touched on prevention as a way to improve adherence to treatment among patients: “When you look at compliance, it’s usually something that’s happening before… [when using PROMs], you’ll probably start to identify things–prevention” (HCP-9). It was clear that participants felt that there was a potential role for PROMs in clinical care to capture different domains of well-being and QOL.

### The role of PROMs to improve pediatric patient communication and engagement

The usefulness of PROMs to improve communication within patient care was a predominant view among participants. This is encouraging as several patients commented on their lack of engagement in the current healthcare conversation: “Usually, I just leave Mommy to do all the talking” (PT-4); “I don’t really get anything from it” (PT-2). A caregiver reiterated a similar view: “My kids, they just don’t even think of the medical practitioners as talking to them. My guess would be that if you asked them, they would say that the appointment was for me” (CG-2).

HCPs acknowledged that PROMs could enhance communication within various dyads, including patients and HCPs, patients and caregivers, caregivers and HCPs, as well as amongst teams of HCPs. Further, it was noteworthy that all participants identified the priority of capturing the patients’ perspective using PROMs. HCPs, in particular, highlighted the role for PROMs to give priority to the patient voice: “[A patient] would feel like their voice is being heard and they’re actually having a chance to say what they think and what they feel because you don’t always have a chance to do that in clinic” (HCP-10). HCPs also identified the importance of receiving information directly from the patient about their preferences for care. As one HCP stated: “I'm always stunned at the assumptions we make that are totally wrong about what patients want” (HCP-2). Another HCP summarized:

I do think it would provide us with information that we may not be getting. Especially when the dynamics – when there’s a parent in the room and there’s a child – you might be getting… what’s important to the parent as opposed to what’s important to the child. [PROMs] may help highlight them to us” (HCP-4).

Patients identified several other potential benefits to using PROMs. One patient noted the ability to safely disclose concerns and feelings when communicating with HCPs using PROMs: “I can talk about the worries I have without having to bring it up myself” (PT-5). Another patient described sharing information related to QOL as beneficial because it would provide a preferred alternative to “just keeping it inside” (PT-3). A few participants also highlighted the value of proxy reports, particularly if patients were hesitant to share: “I think it would be great for parents to fill out… ‘cause the patient could be like, ‘everything’s great’ but the parent could be like ‘actually… [my child is] doing this’ and you can discuss it” (PT-9).

Caregivers acknowledged that patients’ willingness to complete PROMs would depend on the individual child. For example, one parent identified their child as an introvert: “I mean, my child’s an introvert. So, I think sometimes [disclosing] is really uncomfortable for [them]” (CG-5). Another parent suggested that a child may not be able to see the long-term or immediate benefit of completing PROMs and that self-reporting may be a new consideration: “I think it’s a big challenge [getting patients to complete PROMs] ‘cause they might not see the benefit of it yet… it might be an adjustment for them” (CG-6).

Collectively, caregivers highlighted that using PROMs in clinical care could lead to improved patient engagement: “I can see that [PROMs] would have [my child] instantly more involved in [their] own healthcare” (CG-3). Patients expressed the same potential: “[PROMs] can help you feel like you [are] part of the [healthcare] team” (PT-6). Some caregivers and HCPs identified that PROMs could be “a really good transition tool” (CG-4) when pediatric patients transition to adult care, which could facilitate improved health outcomes. In a similar context, a few HCPs explained: “If you can get the kid to participate, you’re going to have better cooperation” (HCP-4); “If patients are more engaged, their outcomes will improve, and you’ll see that” (HCP-6).

### The role of PROMs to inform clinical pediatric transplant care

The responsibility of HCPs to respond promptly to patients’ PROM data emerged as a dominant concern across caregiver and HCP participants. Caregivers vocalized previous experiences of providing information to HCPs with “no follow up” (CG-7)*.* HCPs also recognized the importance of timely follow up and further indicated the value of having “a very clear protocol” (HCP-11) to ensure consistency in care across patients.

Caregivers and patients supported a protocol that allowed all HCPs on a patient’s team to access the PROM data to enable a review prior to clinic visits. This would facilitate an opportunity to discuss PROM results with patients during clinic. While the majority of caregivers expressed support for patients to review PROM results “away from mom and dad” (CG-8) and independently with a HCP, most patients were open to the presence of their parents/caregivers during the review process: “I think it’s good [for parents to be present] so the parent knows how the patient thinks that they’re doing” (PT-3). Many patients, caregivers, and HCPs also spoke about patients’ strong relationships with nurses who might be “a good fit” (PT-2) as a consistent HCP to review PROM results with patients.

Participating HCPs felt that reviewing PROM data should be used as a “screening measure” (HCP-8) or a “starting point for conversation” (HCP-12) and not as a diagnostic measure. One HCP stated: “I think we need to be cautious [about] interpretation, 100 percent. It has to be a conversation” (HCP-5). Ensuring the accurate interpretation of data was important to several HCPs, as reflected by one participant: “It’s a mental health screening [tool]–not a full assessment–and what we might be picking up on… symptoms of depression or elevated anxiety scores–mak[ing] sure that it’s accurately represented” (HCP-7). Involving allied HCPs, such as social workers or psychologists, was identified as a critical resource to ensure accurate interpretation of PROM data; a multi-disciplinary team was deemed essential to help determine the need for clinical intervention. All participants noted the importance of ‘patient choice’ if clinical intervention was recommended.

### Insights for effective implementation

#### Instrument selection

Most participants offered positive reviews for both PROMs, the PedsQL™ Generic Core Scales [44] and the PedsQL™ Transplant Module [45]. However, the PedsQL™ Transplant Module [45] was preferred by most, noting that the questions were more relevant to the transplant experience: “It’s a lot more relatable to transplant patients” (PT-1); “I think that it fits [patients’] world a little better” (HCP-1).

#### Electronic or paper-based administration

The majority of participants vocalized a strong preference for electronic administration and scoring of PROMs. In particular, participants cited multiple benefits of electronic administration, including patients’ ability to complete PROMs remotely and prior to clinical appointments, as well as HCPs having access to PROM data in ‘real-time’ to review and identify potential issues before meeting with patients at their next visit. Participants noted that timely feedback was a key priority to support compliance and meaningful engagement using PROM data. Those who expressed an interest in completing paper-based PROMs (7/63) were not opposed to electronic implementation. Patients, caregivers and HCPs frequently noted the common use of electronic devices among patients, and thus, support for electronic integration was praised across participants: “I think kids would like to do it on the computer” (PT-6); “I would love that it’d be online” (CG-9).

#### Patient assessment eligibility

To determine which patients should complete PROMs, participants considered both age and cognitive ability. Participants acknowledged a consideration for cognitive ability to override an age requirement for the completion of PROMs. However, when asked to identify a general age range to introduce PROMs, most participants highlighted ages eight to 10 years old, stating: “Some kids mature differently than other kids so, I think maybe eight to 10” (PT-4); “If they’re filling it out themselves then probably around eight” (HCP-10).

#### Setting for PROM completion

Patients, caregivers and HCPs endorsed the administration and completion of PROMs to take place remotely and prior to scheduled clinical encounters. Numerous advantages were discussed by participants, including the flexibility of timing: “You can do it whenever you have free time cause when you come to clinic you have to plan it out and everything” (PT-5). In addition, many participants spoke about preferring to complete PROMs outside clinic, given its chaotic atmosphere. One caregiver noted: “Filling it out before would be better–you can do it at your own leisure, whereas [in] clinic, it’s so rushed” (CG-1). Healthcare practitioners were also in agreement of completing PROMs remotely: “Doing it pre-clinic and then discussing it in the clinic makes a whole lot more sense. It’s easier for me to see the clinical value in that” (HCP-7).

#### Frequency of administration

There was a lack of consensus on how often patients should be asked to complete PROMs. A number of factors were discussed related to patient well-being and logistics of pre-scheduled clinic appointments. A patient’s pre- or post-transplant status and associated level of well-being were considered by most participants. Some participants were supportive of pre-transplant administration to “help pave the way… for a successful transplant” (CG-3). Caregivers and HCPs highlighted an unpredictable period of recovery immediately post-transplant, and many participants supported PROMs administration once patients were a minimum of “three months post-transplant” (HCP-1). Burden on patients and families was an additional factor of consideration, in terms of the length of PROMs and its frequency of completion. The frequency of participating patients’ clinical follow up varied from once a month to once a year. While some participants agreed with syncing PROM administration with every clinical appointment, others were supportive of a standard frequency across all patients, such as “every six months” (PT-1) or “once a year” (PT-8).

## Discussion

This study contributes to current research that supports the use of PROMs in clinical pediatric care and guides their effective implementation into practice. Participant stakeholders (patients, caregivers and HCPs) supported the use of PROMs in pediatric transplant care and described their role as encouraging discussions about patient well-being, improving patient engagement, and informing their clinical care. In particular, HCPs emphasized the potential role of PROMs to proactively address mental health, highlighting the potential negative psychological and social impacts associated with pediatric transplantation [23–29]. Pediatric transplant patients noted their lack of engagement or direct communication with HCPs, offering almost unanimous support for the collection of PROMs to share their perspectives and safely disclose concerns during clinic visits.

Existing literature in both pediatric and adult chronic illness populations indicates improved patient-provider communication with the aid of PROMs, in particular, giving attention to patient psychosocial concerns that are often overlooked in clinic [12, 19]. HCPs in the current study reported a similar perspective and there was consensus among all stakeholders that capturing the patient’s voice should be a priority in clinical care.

Most participants preferred the use of a disease-specific PROM (PedsQL™ Transplant Module [38]) as a means to share patient experience. Further, participants indicated that eight to 10 years of age was ideal to initiate PROMs assessments; current research shows that most children are able to respond reliably to PROMs at this age [44]. While some participants acknowledged a role for caregivers to complete self- or proxy-reported PROMs (of their child’s experience), researchers have identified discrepancies between child self-report and parent proxy-report, supporting the view that proxy-reporting should not be used as a substitute for child self-report [45–47].

The use of PROMs in clinical practice is quickly moving towards electronic administration in both pediatric and adult healthcare settings [15, 18, 39, 40]. An evaluation of the pediatric, Dutch ePROM system, KLIK, revealed that HCPs found ePROM data useful in 95%–100% of clinical consultations [18]. One benefit of using an ePROM platform in a pediatric setting is the cross-compatibility of electronic devices (e.g., laptop, smartphone, tablet) which could cater to the widespread Internet use among children and youth [48]. In the current study, all HCPs and a majority of patients and caregivers supported the use of an ePROM platform to collect and integrate PROM data in a pediatric transplant clinical care setting. Bennett, Jensen and Basch [49] reviewed five ePROM systems used in adult oncology and concluded that ePROM platforms provided instantly accessible data, eliminated manual data entry and scoring, created easily interpretable reports and saved time during clinic visits. In comparison, paper-based PROMs have been cited as a major barrier to integrating PROM data into clinical care with the increased time and human resource capacity required to collect, analyze, and report results [49].

In the current study, caregivers and HCPs believed that prompt feedback to patients and families to inform clinical pediatric transplant care was best facilitated by an ePROM system in comparison to paper-based PROM administration. Further to administrative advantages, eHealth technology provides innovative approaches to understand children’s perspectives of their illness experience [49–52]. Our findings suggest that integrating ePROMs into clinical settings may support a system of care that proactively screens for psychosocial concerns in this patient population. Future research will need to explore if ePROMs are associated with improved health outcomes in clinical pediatric transplant care, such as QOL and medication adherence.

Limitations of this study include recruiting only English-speaking participants as well as those without severe cognitive impairment. It is possible that the perspectives of these individuals may be different from the current findings. Further, while PROM instruments and an e-platform were presented as exemplars, the specific instruments and platform reviewed may have influenced participants’ perceptions. Future studies will need to explore how to ensure PROMs are accessible in the face of language barriers and cognitive challenges to allow for accurate self-report. They will also need to incorporate various PROM measures to facilitate the cross-comparison of instruments. While the disease-specific PROM (PedsQL™ Transplant Module [38]) may be preferred to elicit patient experience, the benefit of using generic instruments (e.g., PedsQL™ Generic Core Scales [37]) to facilitate direct comparisons across disease groups, interventions, treatment regimens, and population norms cannot be overlooked when planning future research protocols [53, 54].

The best-practice methods to implement ePROMs into pediatric clinical practice are not universally established. Consensus will require further collaboration and support to address individual, organizational, community, and contextual factors of implementation [32, 33]. Most recently, the International Society for the Quality of Life (ISOQOL) published two reports highlighting important methodological and operational decisions for the clinical implementation of PROMs [55, 56]. The current study captured the perspectives of patients, caregivers and HCPs in a pediatric transplant setting about the role of PROMs in clinical care and specific considerations for its implementation. The diversity of clinical protocols across pediatric transplant programs and centres in Canada will need to be considered to move towards a consensus of best-practice for ePROMs in pediatric clinical care. Further, considerations for professional development, HCP training, and patient-family education are essential. Next steps should include the development, usability testing, and evaluation of a proposed ePROM platform which could inform future research initiatives.

## Data Availability

Research data (interview transcripts) cannot be shared.

## Abbreviations

ePROM: Electronic patient-reported outcome measure
HCP: Healthcare practitioner
PROM: Patient-reported outcome measure
QOL: Quality of life
